# Association of the new zero-tolerance drinking and driving law with hospitalization rate due to road traffic injuries in Brazil

**DOI:** 10.1038/s41598-022-09300-y

**Published:** 2022-03-31

**Authors:** Cássia Rebeca de Lima Souza, Letícia Xander Russo, Everton Nunes da Silva

**Affiliations:** 1 Postgraduate Program for Collective Health, University of Brasilia, Campus Universitário Darcy Ribeiro, Brasília, DF 70910-900 Brazil; 2 Department of Economics, Federal University of Grande Dourados, Dourados, Brazil; 3 Faculty of Ceilândia, University of Brasilia, Brasília, Brazil

**Keywords:** Health care economics, Health policy, Public health

## Abstract

We investigated the association of the new zero-tolerance drinking and driving law (Law 12,760, Dec. 2012) with hospital admissions due to road traffic injuries in Brazil by using interrupted time series from 2008 to 2019. We used linear regression designed to adjust for autocorrelation and Cumby–Huizinga test for residual autocorrelation. Newey–West standard errors was used to handle heteroscedasticity. We used ICD-10 codes for land transport accidents (V01–V89). The hospitalization rate was calculated per 100,000 inhabitants. The sources were the Hospital Information System and the Brazilian Institute for Geography and Statistics. Pre- and postintervention consist of 59 and 85 months, respectively. For Brazil, the hospitalization rate was associated with a reduction of 0.34 (*p* = 0.097; 95% CI − 0.74 to 0.06) in the first month of the intervention (Dec. 2012), followed by a significant change in the hospitalization trend. Compared to the period prior to the intervention, the monthly trend was associated with a reduction of 0.05 (*p* < 0.01; 95% CI − 0.06 to − 0.04) in the post period. These results stand in agreement with subgroup analyses for the Brazilian regions, although North and Northeast regions did not immediately reduce hospitalization rates (level change). Our results suggested that 440,599 hospitalizations for land transport accidents would be averted by the new zero-tolerance drinking and driving law from Dec. 2012 to Dec. 2019 in Brazil. Even using a quasi-experimental approach, our findings must be interpreted with caution due to observational design and registration flaws surrounding our data.

## Introduction

Road traffic injuries are a global health problem, particularly for males, young people and those living in low- and middle-income countries^1–4^. To achieve the target of reducing road traffic deaths and injuries based on the United Nations (UN) Sustainable Development Goals by 2030^5^, the UN has urged local governments to adopt multiple strategies, including enforcement of traffic laws^6,7^. According to the World Health Organization, 176 countries reported having a drink-driving law at the national level^8^. When these laws are combined with visible and rapid enforcement, they seem to effectively reduce alcohol-related crashes and deaths^9,10^.

Since 19th June 2008, Brazil has adopted a zero-tolerance drinking and driving law, by which motor vehicle drivers under any influence of alcohol are subjected to penalties such as larger fines, longer licence suspension, vehicle seizure and imprisonment^11^. However, under Law 12,760, from 20th December 2012, zero-tolerance drinking and driving law reached its higher effectiveness in terms of enforcement, particularly by increasing the use of sobriety checkpoints and including other evidence to prove driver’s intoxication (driver’s appearance and actions at the scene). There is evidence on zero-tolerance drinking and driving law on mortality rate due to road traffic injuries^12–14^, but not on hospitalization rate. Moreover, these studies also relied on the effect of the first zero-tolerance drinking and driving law (Law 11,705). Investigating the avert hospitalizations due to stricter drink driving law (if any) can lead to some light on the potential benefit to the health system, such as reallocation of hospital beds to other causes and reduction of hospital expenditures owing to road traffic injuries.

Our study aimed to first estimate the association of the new zero-tolerance drinking and driving law (Law 12,760) with hospitalization rate due to road traffic injuries in Brazil by using a quasi-experimental approach through interrupted time series from 2008 to 2019. Then, we used these estimates to predict the averted hospitalizations owing to the new zero-tolerance drinking and driving law from December 2012 to December 2019.

## Methods

### Study setting

Brazil ranks fifth in the world in total area (8.51 million km^2^) and sixth in population size (212 million inhabitants)^15^. In 2019, Brazil had 75,800 and 10,400 km of paved and unpaved roads, respectively^16^. There were 107,948,371 vehicles operating on roads across the country in 2020, with cars and motorcycles representing 58.74% and 22.10%, respectively^17^. Since 1988, Brazil has provided universal healthcare coverage free of charge at the point of service through the Unified Health System (SUS, acronym in Portuguese). SUS delivers primary and specialty care, including health promotion and prevention, diagnosis, and treatment at any lifespan need^18^. Approximately 75% of the Brazilian population has access to healthcare only through SUS, since 25% have private health insurance^19^. Brazil has 5570 municipalities and approximately 85% of the Brazilian population live in urban areas. It is an upper middle-income country with GDP per capita of $ 14,059 in 2020 (adjusted by purchasing power parity—PPP), but with high income inequality across de country.

### Introduction of laws against drinking and driving in Brazil

The first attempt to regulate drinking and driving in Brazil was in 1997, with Law 9503 from the 23rd of September 1997, by which the Brazilian Traffic Code (*Código de Trânsito Brasileiro*) was created. Under this law, the blood alcohol concentration (BAC) limit was 6 dg/L. Levels higher than this limit would result in fine vehicle retention, driver licence suspension, and imprisonment. However, the effectiveness of law was low due to systematic failure in enforcing the existing legislation in terms of inspection and application of punishments to offenders^14^. In 2008, it enacted the zero-tolerance drinking and driving law (*Lei Seca*), Law 11,705, from the 19th of June 2008, lowering the BAC limit to 0.0 dg/L and adding the suspension of the driver’s licence for 12 months, imprisonment for blood alcohol concentration over 6 dg/L, fine, and vehicle retention whether the driver’s BAC was over the legal limit^12^. To test intoxication, the traffic agent may ask the driver to perform a breath test (breathalyzer) or a blood test. However, the Justice assured the right of drivers to refuse a breathalyzer test based on the Pact of San Jose, invoking “the right not to be compelled to be a witness against himself or to plead guilty”, which substantially reduced the law’s effectivity^20–22^. In 2012, Brazil adopted a hard-line stance against those caught driving under the influence of alcohol above the legal limit, called the new zero-tolerance drinking and driving law. Law 12,760 was enacted on the 20th of December 2012, which keeps the previous restrictions (BAC limit to 0.0 dg/L, suspension of driver licence for 12 months, imprisonment, fine [increased by twofold], and vehicle retention). However, this law has also included the officer’s observations in the report as evidence of intoxication, i.e., the driver’s appearance and actions at the scene can also be used as concrete evidence of intoxication. Other evidence has also been used, such as witnesses and videos. After 2012, there was also an increasing use of sobriety checkpoints to identify drinking and driving by systematically and randomly stopping drivers for the assessment of alcohol impairment. This strategy has also increased the perceived risk of arrest for alcohol-impaired driving^23^.

There are other challenges related to the enforcement of drinking and driving laws in Brazil besides the ones reported above. The alcoholic beverage industries have strong influence in the economic and political arenas. This sector represents around 3% of the Brazilian GDP and beer is classified as cold drinks (as soft drinks and sports drinks), which means that it has a lower taxation than other alcoholic drinks^24^. Brazil has also faced struggles with illegal consumption of alcoholic beverages by adolescents as many commercial establishments fail to ask adolescents for their identity documents^24^. This consumption is also fostered by alcohol advertisements on TV and other medias^25^.

Table 1 shows the main characterization for each drinking and driving law.

**Table 1:** Characteristics of the main regulation on drinking and driving law in Brazil. *Source*: Brazilian Traffic Code^26^. Note: We used the reference value currently in effect in Brazil (R$ 293.47) and the 2019 purchasing power parity—PPP—conversion factor of 2.247 from the World Bank.

Characterization	Law 9508 1997	Law 11,705 2008	Law 12,760 2012
Blood alcohol concentration	> 6 dg/L	Any blood alcohol level	Any blood or breath alcohol level
Penalty	Considered a very-serious infraction + Fine of fivefold the reference-value + Temporary driver's licence suspension (without defining the period of suspension) + Vehicle retention	Considered a very-serious infraction + Fine of fivefold the reference-value + Driver's licence suspension by 12 months + Vehicle retention	Considered a very-serious infraction + Fine of tenfold the reference-value + Driver's licence suspension by 12 months + Vehicle retention
Fine	R$ 1,467.35 (= Int$ 653.03)	R$ 1,467.35 (= Int$ 653.03)	R$ 2,934.70 (= Int$ 1,306.05)
Prison	Imprisonment from 6 to 36 months, since it was proved that the driver was under influence of alcohol and posed a significant threat to others. It was hard to prove it based on the law	Imprisonment from 6 to 36 months for blood alcohol concentration over 6dg/L. Driver could refuse to be tested	Imprisonment from 6 to 36 months for blood alcohol concentration over than 6dg/L or breath alcohol concentration over than 0.3 mg/L. Whether the driver refused to be tested, other evidence can be used (witness, videos, etc.)

### Variables and sources

Our variable of interest is the rate of hospitalization for land transport accidents per 100,000 inhabitants. We used the Hospital Information System (SIH/SUS)^27^ and the Brazilian Institute of Geography and Statistics (IBGE)^15^ to collect monthly data on hospitalizations and population size, respectively. We used the 10th revision of the International Classification of Diseases (ICD-10) codes for external causes of morbidity related to land transport accidents (V01–V89). The rate of hospitalization was calculated for Brazil, but we also stratified this variable by Brazilian region (north, northeast, middle-west, southeast and south), sex (male and female), and age group (0–4; 5–9; 10–14; 15–19; 20–29; 30–39; 40–49; 50–59; 60–69; 70–79; and 80 +). The Ministry of Health maintains the SIH/SUS, by which all hospital admissions delivered by the public health system across the county are recording and reimbursed. SIH/SUS has been recognised as a relevant source of data to support healthcare planning and management, constituting a single comprehensive source for public hospital admissions at national level, regardless its registration flaws^28,29^.

### Statistical analyses

We used interrupted time series to estimate the association of new zero-tolerance drinking and driving laws with the rate of hospitalizations for land transport accidents in Brazil. Interrupted time series analysis is a quasi-experimental approach by which associations are estimated based on the pre- and postintervention periods using regression modelling, by using the formula below^30^

$$Y_{t} = \beta_{0} + \beta_{1} \times time_{t} + \beta_{2} \times intervention_{t} + \beta_{3} \times time\; after\; intervention_{t} + e_{t}$$

where intervention_t_ = level. Time after intervention_t_ = trend.

Pre- and postintervention were defined based on the period before (Jan/2008–Nov/2012) and after exposure to Law 12,760 was enacted (Dec/2012–Dec/2019), with data frequency per month. This resulted in 59 months before the beginning of the intervention and 85 months after the intervention. The series was adjusted for seasonal variation. For that, we employed the Holt–Winters additive method^31^. We also used the Cumby–Huizinga test to investigate residual autocorrelation^32^, by which we identified autocorrelation at lag 4 but not at any higher lag orders (Table S1, supplementary material). Thus, our initial model specifying lag^4^ should correctly account for this autocorrelation. After testing for autocorrelation and considering the lag^4^, we performed the Dickey-Fuller test to investigate the stationarity of the series. The null hypothesis of a unit root was rejected (Z =−3.692; *p* = 0.004). In addition, the Dickey–Fuller was also conducted for the residuals, which showed no evidence of non-stationarity (Z = − 4.944; *p* = 0.00). Moreover, we used ordinary least square regression with Newey–West standard errors to handle autocorrelation and potential heteroskedasticity. The study design assumed a linear time trend. We tested nonlinear patterns by fitting a quadratic model. The quadratic coefficient was not significant, suggesting that the assumption of linearity was appropriate. Excel was used for organizing data and performing descriptive statistics, and STATA 14.2 was used for regression analyses using the ACTEST package.

We also calculated the averted hospitalizations for land transport accidents associated with the introduction of the new zero-tolerance drinking and driving law. We used the initial estimated trend to forecast the number of hospitalizations in a scenario of non-intervention (Law 12,760) from November 2012 to December 2019. The averted hospitalizations were calculated by subtracting the forecast values from the predicted values (model considering the intervention). To calculate the hospitalization cost averted by the law, we considered the average reimbursement fees for hospitalization cause-related to land transport accident in 2019 (R$ 1,263.46) and the 2019 purchasing power parity—PPP—conversion factor of 2.247 from the World Bank. To calculate the hospital days averted, we considered the 2008–2019 average length of stay of 6.1 days per hospitalization.

### Sensitivity analyses

We also performed sensitivity analyses on the starting point of our time series and our exposure month. The zero-tolerance drinking and driving law (*Lei Seca*), Law 11,705, was introduced in June 2008. Based on that, the first five months of our time series (January and May 2008) were excluded to investigate their possible influence on the results. As interrupted time series rely on ordinary least squares (OLS), which minimizes the sum of squared residuals^33^, the estimates are sensitive to outliers. We dropped the first five months and considered an alternative period (from June/2008 to Nov/2019). In addition, although Law 12,760 was enacted on 20th December 2012, it could have some delay in terms of its implementation. To take this scenario into account, we considered the implementation of the law in January or February 2013. We also combined the two sensitivity analyses.

### Ethics clearance

We used secondary and publicly available data, whose information was aggregated and had no possibility to identify any individual. Based on that, this study did not have to be submitted to the Research Ethics Committee, in accordance with Resolution 510/16 of the Brazilian National Health Council^34^.

## Results

**Figure 1:**
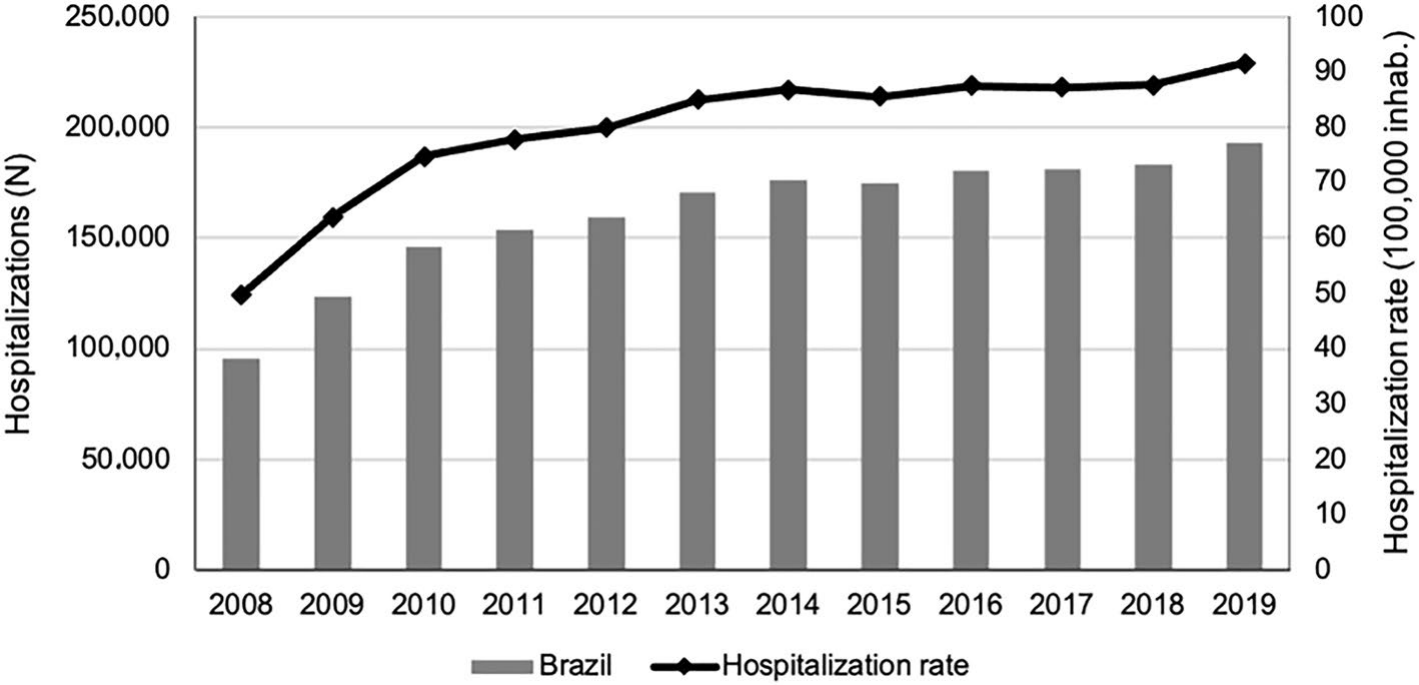
Trend in hospitalizations for land transport accidents (absolute and rate per 100,000 inhabitants) from 2008 to 2019, Brazil.

In the period analysed (2008–2019), SUS performed 12,566,104 hospitalizations for external causes. Of them, 1,937,064 (15%) were cause-related to land transport accidents, with an average of 13,453 per month. The hospitalization rate for land transport accidents increased from 49.7 to 91.5 per 100,000 inhabitants between 2008 and 2019 (Fig. 1).

All age groups increased the hospitalization rate for land transport accidents from 2008 to 2019. People from the 20–29 age group ranked first in the hospitalization rate for land transit accidents during the whole period investigated. However, the 40–49 age group presented the highest growth, with an increase of 114.1% between 2008 and 2019 (Table 2).

**Table 2:** Hospitalization rate for land transport accidents stratified by age group from 2008 to 2019, Brazil.

Age group	Year																								Growth 2008–2019 (%)
	2008		2009		2010		2011		2012		2013		2014		2015		2016		2017		2018		2019		
	N	Rate	N	Rate	N	Rate	N	Rate	N	Rate	N	Rate	N	Rate	N	Rate	N	Rate	N	Rate	N	Rate	N	Rate	
0–4	2068	12.7	2835	17.7	2910	18.4	2837	18.2	2811	18.3	3089	20.4	2822	18.9	2494	16.9	2367	16.3	2417	16.8	2259	15.9	2135	15.2	20.0
5–9	3986	23.2	5144	30.1	5404	31.9	5152	30.9	5178	31.4	5104	31.4	4659	29.1	4286	27.2	4033	25.9	3788	24.7	3564	23.6	3649	24.5	5.4
10–14	4820	28.0	6006	34.9	6622	38.5	6740	39.1	6731	39.1	7031	41.0	6695	39.3	6206	36.7	5928	35.6	5823	35.4	5353	33.0	5091	31.8	13.6
15–19	10,792	62.7	13,389	77.9	15,382	89.6	17,231	100.5	18,057	105.3	19,543	114.0	20,368	118.8	19,750	115.2	19,608	114.2	19,310	112.6	18,012	105.4	17,875	105.2	68.0
20–29	29,233	83.4	37,655	107.2	44,619	127.1	46,235	132.0	46,321	132.9	48,254	139.4	49,170	142.9	48,899	142.8	50,833	149.0	50,985	149.8	51,022	150.0	54,185	159.4	91.0
30–39	17,296	59.7	22,924	77.6	28,791	95.5	30,463	98.8	31,775	100.7	34,713	107.5	36,413	110.5	36,713	109.6	38,601	113.8	38,288	111.9	39,741	115.5	41,246	119.5	100.2
40–49	11,961	49.3	15,858	64.2	19,242	76.8	20,463	80.5	21,650	84.2	23,771	91.3	24,771	94.0	25,312	94.6	26,576	97.8	26,620	96.3	27,834	98.8	30,328	105.5	114.1
50–59	7102	41.3	9604	53.9	11,467	62.2	12,509	65.6	13,413	68.1	14,800	72.8	16,167	77.3	16,421	76.4	17,307	78.6	18,020	80.1	19,234	83.8	20,352	87.1	110.9
60–69	4,081	40.3	5235	49.7	6157	56.1	6480	56.6	7348	61.4	8080	64.6	8348	63.8	8299	60.8	8852	62.3	9391	63.5	9599	62.5	10,605	66.5	65.0
70–79	2562	45.7	3174	54.9	3572	60.0	3708	60.4	4034	63.8	4278	65.6	4381	65.0	4417	63.2	4322	59.5	4381	58.0	4676	59.3	4982	60.5	32.3
80+	1261	51.7	1679	65.6	1900	71.0	1814	64.9	1898	65.1	2142	70.5	2213	69.8	2036	61.5	2016	58.3	2111	58.3	2156	56.9	2365	59.7	15.6

Males accounted for approximately 73% of all hospitalizations cause-related to land transport accidents in the period analysed. From 2008 to 2019, both sexes presented an increase of approximately 85% in the hospitalization rate per 100,000 inhabitants, taking into account the entire county (Table 3).

**Table 3:** Hospitalizations (absolute and rate) for land transport accidents stratified by sex from 2008 to 2019, Brazil.

Year	Men		Woman	
	N	Rate	N	Rate
2008	74,796	78.9	20,366	21.1
2009	96,074	100.3	27,429	28.1
2010	114,406	118.3	31,660	32.0
2011	120,487	123.4	33,145	33.2
2012	124,778	126.7	34,438	34.2
2013	133,562	134.5	37,243	36.6
2014	137,924	137.7	38,083	37.1
2015	137,062	135.8	37,771	36.5
2016	142,007	139.6	38,436	36.8
2017	141,801	138.4	39,333	37.4
2018	143,407	139.0	40,043	37.8
2019	151,102	145.5	41,711	39.1

**Figure 2:**
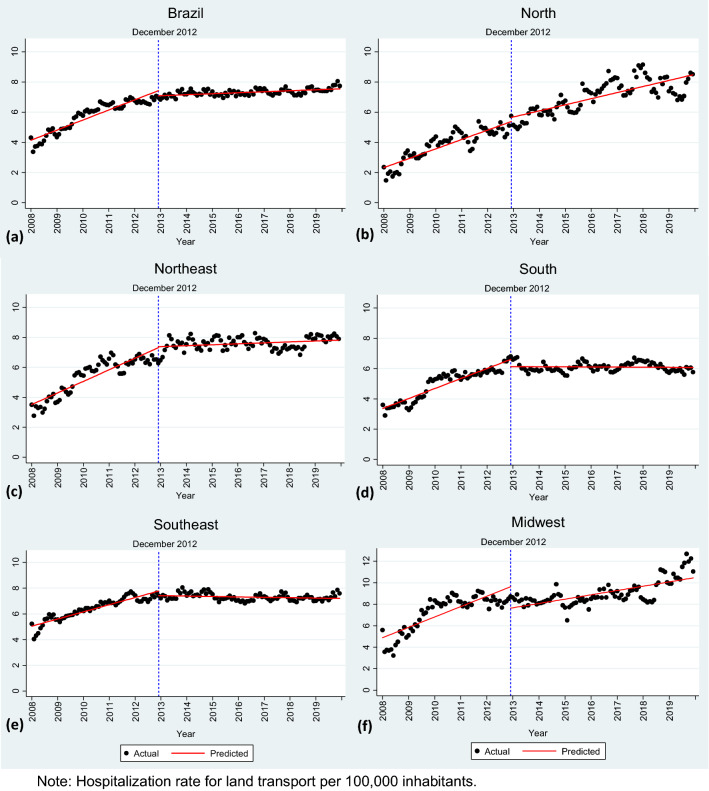
Graphical representation of the new zero-tolerance drinking and driving law on hospital rate for land transport accidents from interrupted time series regression for Brazil (**a**) and its regions (**b**–**f**), 2008–2019.

Table 4 shows the regression estimates for the hospitalization rate for land transport accidents per 100,000 inhabitants in Brazil and its regions. For Brazil, the initial hospitalization rate was estimated at 4.09 per 100,000 inhabitants (January 2008). In the period prior to the introduction of the new zero-tolerance drinking and driving law, the trend sharply increased, with an increase of 0.06 per 100,000 inhabitants per month (*p* < 0.01; 95% CI 0.04–0.07). After the intervention was implemented (December 2012), the hospitalization rate was associated with a reduction of 0.34 per 100,000 inhabitants in the first month (*p* = 0.097; 95% CI − 0.74 to 0.06). In the following months, the monthly trend of hospitalization rate was associated with an increase of 0.0055 (*p* < 0.01; 95% CI 0.0029–0.0081), indicating a weaker upward trend (or − 0.05 in relation to the trend in the period prior to the intervention). Figure 2a shows the hospitalization rate for land transport accidents before and after the intervention.

**Table 4:** Results from the interrupted time series regression for Brazil and regions, 2008–2019.

	Brazil	North	Northeast	South	Southeast	Midwest
Time	0.06***	0.05***	0.06***	0.05***	0.05***	0.08***
	(0.04–0.07)	(0.04–0.06)	(0.05–0.08)	(0.05–0.06)	(0.04–0.06)	(0.05–0.11)
Level	− 0.34*	0.27	0.07	− 0.47**	− 0.36*	− 2.00***
	(− 0.74 to 0.06)	(− 0.31 to 0.86)	(− 0.47 to 0.60)	(− 0.86 to − 0.09)	(− 0.78 to 0.05)	(− 3.19 to − 0.81)
Trend	− 0.05***	− 0.02**	− 0.06***	− 0.06***	− 0.05***	− 0.05***
	(− 0.06 to − 0.04)	(− 0.04 to − 0.00)	(− 0.07 to − 0.04)	(− 0.07 to − 0.05)	(− 0.06 to − 0.04)	(− 0.08 to − 0.01)
Constant	4.09***	2.27***	3.46***	3.31***	5.00***	4.81***
	(3.70–4.47)	(1.80–2.75)	(2.99–3.93)	(2.97–3.66)	(4.63–5.38)	(3.73–5.90)
Observations	144	144	144	144	144	144

95% CI in parentheses.

***p < 0.01, **p < 0.05, *p < 0.1.

The new zero-tolerance drinking and driving law seems to play a similar role in the mid-west, southeast and south regions as it did in Brazil as a whole (Fig. 2a,d,e,f). In the north and northeast, we did not identify a significant reduction in the hospitalization rate immediately after Law 12,760 was enacted. Although there was no change in the hospitalization level, the monthly trend coefficient was statistically significant and negative, suggesting a weaker upward trend after the new zero-tolerance drinking and driving law (Fig. 2b,c).

Table 5 reports the results by sex and age group. We found that Law 12,760 was associated with an immediately reduction on hospitalization rate for young men (15–39 years old). In terms of trend, all groups showed a statistically significant reduction in monthly rate after the intervention, when compared with the period prior to the intervention.

**Table 5:** Results from the interrupted time series by sex and age group, Brazil, 2008–2019.

	Sex					
	Men	Woman				
Time	0.09***	0.02***				
	(0.07–0.11)	(0.02–0.03)				
Level	− 0.56*	− 0.13				
	(− 1.21 to 0.09)	(− 0.31 to 0.06)				
Trend	− 0.08***	− 0.02***				
	(− 0.10 to − 0.06)	(− 0.03 to − 0.02)				
Constant	6.44***	1.77***				
	(5.84–7.05)	(1.58–1.96)				
Observations	144	144				

	Age group					
	0–14 years	15–29 years	30–39 years	40–49 years	50–59 years	60 + years
Time	0.01***	0.09***	0.08***	0.06***	0.05***	0.03***
	(0.01–0.02)	(0.07–0.11)	(0.06–0.09)	(0.05–0.08)	(0.04–0.06)	(0.02–0.04)
Level	− 0.07	− 0.57*	− 0.64**	− 0.28	− 0.26	− 0.21
	(− 0.29 to 0.16)	(− 1.17 to 0.03)	(− 1.25 to − 0.02)	(− 0.72 to 0.16)	(− 0.63 to 0.11)	(− 0.60 to 0.19)
Trend	− 0.02***	− 0.08***	− 0.06***	− 0.05***	− 0.03***	− 0.04***
	(− 0.03 to − 0.02)	(− 0.10 to − 0.07)	(− 0.08 to − 0.05)	(− 0.06 to − 0.04)	(− 0.04 to − 0.02)	(− 0.05 to − 0.02)
Constant	1.90***	6.20***	4.86***	3.99***	3.39***	3.61***
	(1.68–2.13)	(5.69–6.71)	(4.35–5.38)	(3.59–4.39)	(3.01–3.76)	(3.18–4.04)
Observations	144	144	144	144	144	144

95% CIs in parentheses.

***p < 0.01, **p < 0.05, *p < 0.1.

In general, estimates from sensitivity analyses suggested similar results to the base model, except for the coefficient “level” from the North and Northeast regions and groups of women, 0–14 years and over 40 years, which did not reach statistical significance at the 10% level in any specification. North also did not reach statistical significance in the “trend” variable in two specifications (sensitivity analyses 3, 4 and 5) (Supplementary material, Table S2).

Our results suggested that the new zero-tolerance drinking and driving law was associated with 440,599 hospitalizations averted for land transport accidents from Dec. 2012 to Dec. 2019 in Brazil. This means an avoidable cost of Int$ 247.74 million for the same period. Additionally, avoidable hospitalizations would allow the reallocation of 2,687,654 days of hospitalization in SUS in a 7-year period. Taking the worst scenario from the sensitivity analysis, the results would be 341,450 hospitalizations averted, 2,082,845 hospital days averted, and Int$ 191.99 million averted (Table 6). It is worth noting that our results were obtained from an observational study with secondary data.

**Table 6:** Predicted hospitalizations, hospital days and costs averted associated with the new zero-tolerance drinking and driving law, Brazil.

Model	Number of hospitalizations averted	Hospital days averted	Costs averted (Int$, in million PPP)
**Base model**			
Period (Jan/08–Dec/19); exposure (Dec/2012)	440,599	2,687,654	247.74
**Sensitivity analysis 1**			
Period (Jan/08–Dec/19); exposure (Jan/2013)	429,559	2,620,310	241.54
**Sensitivity analysis 2**			
Period (Jan/08–Dec/19); exposure (Feb/2013)	414,692	2,529,621	233.18
**Sensitivity analysis 3**			
Period (Junr/08–Dec/19); exposure (Dec/2012)	377,755	2,304,306	212.41
**Sensitivity analysis 4**			
Period (Jun/08–Dec/19); exposure (Jan/2013)	359,230	2,191,303	201.99
**Sensitivity analysis 5**			
Period (Jun/08–Dec/19); exposure (Feb/2013)	341,450	2,082,845	191.99

## Discussion

Our study suggested that the new zero-tolerance drinking and driving law (Law 12,760) would be statistical associated with a reduction on hospitalization rates for land transport accidents in Brazil. These results stand in agreement with subgroup analyses for the Brazilian regions, although north and northeast regions did not immediately was associated with a reduction on hospitalization rates (level change). Moreover, land transport accidents are more common among young males in both absolute and relative (incidence rate) values. Even using a quasi-experimental approach, our findings must be interpreted with caution due to observational design and registration flaws surrounding our data.

Other studies have also reported worse results for the north and northeast related to mortality for road traffic injuries compared to other regions^35–37^. These results are also in line with data from the National Health Survey undertaken in Brazil in 2019, from which north and northeast presented the highest drinking and driving prevalence among the Brazilian regions, reaching 23.4 and 21.5% of their inhabitants, respectively^38^. Taking the country as a whole, Brazil has reduced the drinking and driving prevalence from 24.4% in 2013 to 17.0% in 2019^38^. Another study showed higher hospitalization rates in the midwest and northeast regions, which probably were associated with low use of safety-equipment in vehicles^39^.

In Brazil, hospitalizations for land transport accidents corresponded to 15.8% of all hospital admissions for external causes in 2011. Males have a 3.8-fold higher chance of being involved in an accident than females^4^. In general, males (27.3%) self-reported having more episodes of driving vehicles after drinking alcohol than women (7.1%), as reported in the Second National Survey on Alcohol and Drugs, 2012^40^.

A similar finding, in 2013, among the victims of land transport accidents undergoing hospitalization in public hospitals or those affiliated with the SUS, there was a predominance of male individuals, young adults and motorcyclists living in the mid-west and northeast regions of Brazil^39^. One possible explanation for this finding is the fact that, culturally, men are more exposed to dangerous situations, such as alcohol consumption and driving a motor vehicle at speeds higher than those allowed on the roads^39^.

In Taiwan, a law that lowered the blood alcohol concentration limit for driving from 0.05 to 0.03 mg/mL was associated with a reduction on the number of drivers under the influence of alcohol, but it did not significantly decrease the associated injuries after the law was imposed^41^. In Japan, a study used time series segmented regression analyses to estimate the effect of a new road traffic law enacted in June 2002, which reduced the blood alcohol concentration level from 0.05 to 0.03 and increased penalties (fines and driver’s license points). Results showed a statistically significant reduction of deaths and injuries^42^.

### Strengths and limitations

We investigated a large time series (144-time units) using a quasi-experimental approach to estimate the association of a new zero-tolerance drinking and driving law with the hospital rate for land transport accidents in Brazil. Most studies have investigated mortality data^43^. To the best of our knowledge, our study is the first attempt to provide estimates of the association of the new zero-tolerance drinking and driving law with hospitalization rate. This outcome is important since it affects costs and available hospital beds in the public health system. We have also relied on a rich administrative database, from which the Ministry of Health reimburses hospital services across the country. Although real-world data are playing an increasing role in evidence-informed decision making, administrative information systems are also affected by registration flaws, which may increase uncertainty about the official statistics and, consequently, our findings. It is worth noting several limitations of our study. First, our estimates refer to hospital admissions in the public health system. Approximately 25% of the Brazilian population has private health insurance. On this basis, our study may underestimate the benefits of the new zero-tolerance law. Second, our preintervention period did not reflect an absence of zero-tolerance drinking and driving law. Law 11,705 enacted in June 2008 had already stated most of the legal penalties that Law 12,760 enacted in December 2012 did. The difference between them is the law’s enforcement, which is much stronger in the latter law. Third, there are several socioeconomic or road safety (poor road infrastructure and/or safety conditions related to vehicles) disparities between and within the Brazilian regions, which may influence drivers’ perceptions of law enforcement. There are also profound differences over time across the country. One example is the option of allowing private companies to charge cars and motor vehicles being driven within highways and roads. Several states have used this strategy to improve the quality of paving, signalling, and safety of roads at different times, particularly São Paulo and other states from the Southeast and South. Four, there are other confounders not considered in our study, such as traffic monitoring, road signalling, and the increasing share of motorcycles over the period investigated in our study. As motorcycles have higher chance to be involved in road traffic accident with injury, they may reduce the potential effect of the new-zero tolerance drinking and driving law since their share is increasing over the period analysed. Fifth, we used observational design and secondary data to estimate our results, which may be prone to incompleteness and under-reporting. However, our data has high external validity, since they correspond to all hospital admissions reimbursed by the Ministry of Health.

### Implications for policies

Since Law 12,760 was enacted in December 2012, Brazil has adopted a hard-line stance against those driving under any influence of alcohol, particularly by increasing the use of sobriety checkpoints and including other evidence to prove drivers’ intoxication (driver’s appearance and actions at the scene). Our estimates indicated that over 400,000 hospitalizations for land transport accidents would be averted by the law during a 7-year period, corresponding to a reduction of Int$ 248 million in inpatient care for the same period. There was also a positive externality to reallocate the hospital days to other health conditions or diseases based on the averted hospitalization for land traffic accidents.

Thinking globally, a stricter drinking and driving law may be a good value for money for low- and middle-income countries, since its implementation appears to be at low cost. However, decision makers must ensure that the law would change drivers’ perception about the legal consequences of being caught driving under any influence of alcohol. For that, local governments must implement frequent and random checkpoints around cities, particularly in areas with a higher incidence of land transport accidents. The media has a central role in disseminating news on the public effort to tackle drinking and driving cases.

## Supplementary Information

Supplementary Information.
