# Supplementary material for: Association of the new zero-tolerance drinking and driving law with hospitalization rate due to road traffic injuries in Brazil
Source: Sci Rep. 2022 Mar 31;12:5447. doi: 10.1038/s41598-022-09300-y (PMC8971401; doi:10.1038/s41598-022-09300-y)
Supplement: Supplementary file 1 — Supplementary Information. [file 41598_2022_9300_MOESM1_ESM.docx]

**Supplementary material**

**Table S1:** Results from the autocorrelation test (Cumby-Huizinga)

| **Lag** | **Chi square** | **Default** | **p-value** | **Lag** | **Chi square** | **Default** | **p-value** |
| --- | --- | --- | --- | --- | --- | --- | --- |
| H0: q=0 (serially uncorrelated)  HA: s.c. present at range specified | | | | H0: q=specified lag-1  HA: s.c. present at lag specified | | | |
| 1-1 | 71,16 | 1 | 0,00 | 1 | 71,16 | 1 | 0,00 |
| 1-2 | 76,56 | 2 | 0,00 | 2 | 29,08 | 1 | 0,00 |
| 1-3 | 80,27 | 3 | 0,00 | 3 | 8,51 | 1 | 0,00 |
| 1-4 | 80,99 | 4 | 0,00 | 4 | 6,08 | 1 | 0,01 |
| 1-5 | 81,36 | 5 | 0,00 | 5 | 1,93 | 1 | 0,16 |
| 1-6 | 82,94 | 6 | 0,00 | 6 | 2,64 | 1 | 0,10 |
| 1-7 | 85,06 | 7 | 0,00 | 7 | 0,35 | 1 | 0,55 |

**Table S2:** Sensitivity analyses performed based on period of the time series and the starting point of the new drinking and driving law by Brazilian regions.

|  |  | **Brazil** | **North** | **Northeast** | **South** | **Southeast** | **Mid-west** |
| --- | --- | --- | --- | --- | --- | --- | --- |
|  |  | **Sensitivity analysis 1: Period (Jan/08 – Dec/19); exposure (Jan/2013)** | | | | | |
| Time |  | 0.05*** | 0.05*** | 0.06*** | 0.06*** | 0.05*** | 0.08*** |
|  |  | (0.04 - 0.07) | (0.04 - 0.06) | (0.05 - 0.08) | (0.05 - 0.06) | (0.03 - 0.06) | (0.05 - 0.11) |
| Level |  | -0.36* | 0.22 | 0.13 | -0.57*** | -0.39* | -2.03*** |
|  |  | (-0.75 - 0.04) | (-0.36 - 0.81) | (-0.38 - 0.64) | (-0.93 - -0.22) | (-0.79 - 0.02) | (-3.19 - -0.88) |
| Trend |  | -0.05*** | -0.02** | -0.06*** | -0.06*** | -0.05*** | -0.04*** |
|  |  | (-0.06 - -0.04) | (-0.04 - -0.00) | (-0.07 - -0.04) | (-0.06 - -0.05) | (-0.06 - -0.04) | (-0.08 - -0.01) |
| Constant |  | 4.10*** | 2.26*** | 3.50*** | 3.31*** | 5.02*** | 4.84*** |
|  |  | (3.71 - 4.49) | (1.80 - 2.72) | (3.01 - 3.99) | (2.97 - 3.65) | (4.65 - 5.38) | (3.76 - 5.93) |
|  |  |  |  |  |  |  |  |
| Observations |  | 144 | 144 | 144 | 144 | 144 | 144 |
|  |  | **Sensitivity analysis 2: Period (Jan/08 – Dec/19); exposure (Feb/2013)** | | | | | |
| Time |  | 0.05*** | 0.05*** | 0.06*** | 0.06*** | 0.04*** | 0.08*** |
|  |  | (0.04 - 0.06) | (0.04 - 0.06) | (0.05 - 0.08) | (0.05 - 0.06) | (0.03 - 0.06) | (0.05 - 0.11) |
| Level |  | -0.35* | 0.25 | 0.17 | -0.65*** | -0.39* | -2.06*** |
|  |  | (-0.74 - 0.04) | (-0.32 - 0.82) | (-0.34 - 0.69) | (-0.99 - -0.31) | (-0.79 - 0.02) | (-3.19 - -0.93) |
| Trend |  | -0.05*** | -0.02** | -0.06*** | -0.05*** | -0.05*** | -0.04** |
|  |  | (-0.06 - -0.04) | (-0.04 - -0.00) | (-0.07 - -0.04) | (-0.06 - -0.05) | (-0.06 - -0.04) | (-0.07 - -0.01) |
| Constant |  | 4.12*** | 2.27*** | 3.53*** | 3.31*** | 5.03*** | 4.88*** |
|  |  | (3.73 - 4.52) | (1.81 - 2.73) | (3.02 - 4.03) | (2.97 - 3.65) | (4.66 - 5.40) | (3.79 - 5.96) |
|  |  |  |  |  |  |  |  |
| Observations |  | 144 | 144 | 144 | 144 | 144 | 144 |
|  |  | **Sensitivity analysis 3: Period (Jun/08 – Dec/19); exposure (Dec/2012)** | | | | | |
| Time |  | 0.06*** | 0.05*** | 0.07*** | 0.06*** | 0.04*** | 0.08*** |
|  |  | (0.04 - 0.07) | (0.04 - 0.07) | (0.05 - 0.08) | (0.04 - 0.07) | (0.03 - 0.05) | (0.05 - 0.12) |
| Level |  | -0.26 | 0.16 | -0.00 | -0.22 | -0.17 | -1.94*** |
|  |  | (-0.59 - 0.08) | (-0.43 - 0.75) | (-0.61 - 0.60) | (-0.63 - 0.18) | (-0.51 - 0.17) | (-3.02 - -0.86) |
| Trend |  | -0.05*** | -0.01 | -0.06*** | -0.06*** | -0.05*** | -0.05*** |
|  |  | (-0.06 - -0.04) | (-0.03 - 0.00) | (-0.08 - -0.04) | (-0.07 - -0.04) | (-0.06 - -0.04) | (-0.09 - -0.02) |
| Constant |  | 4.45*** | 2.67*** | 3.81*** | 3.61*** | 5.40*** | 5.35*** |
|  |  | (4.13 - 4.77) | (2.23 - 3.10) | (3.34 - 4.28) | (3.16 - 4.05) | (5.19 - 5.60) | (4.34 - 6.36) |
|  |  |  |  |  |  |  |  |
| Observations |  | 139 | 139 | 139 | 139 | 139 | 139 |
|  |  | **Sensitivity analysis 4: Period (Jun/08 – Dec/19); exposure (Jan/2013)** | | | | | |
| Time |  | 0.05*** | 0.05*** | 0.07*** | 0.05*** | 0.04*** | 0.08*** |
|  |  | (0.04 - 0.07) | (0.04 - 0.06) | (0.05 - 0.08) | (0.04 - 0.07) | (0.03 - 0.05) | (0.05 - 0.11) |
| Level |  | -0.23 | 0.14 | 0.03 | -0.22 | -0.14 | -1.93*** |
|  |  | (-0.58 - 0.12) | (-0.45 - 0.72) | (-0.56 - 0.62) | (-0.64 - 0.20) | (-0.51 - 0.23) | (-3.00 - -0.85) |
| Trend |  | -0.05*** | -0.01 | -0.06*** | -0.06*** | -0.04*** | -0.05*** |
|  |  | (-0.06 - -0.04) | (-0.03 - 0.00) | (-0.08 - -0.04) | (-0.07 - -0.04) | (-0.06 - -0.03) | (-0.09 - -0.01) |
| Constant |  | 4.48*** | 2.66*** | 3.84*** | 3.63*** | 5.42*** | 5.40*** |
|  |  | (4.14 - 4.81) | (2.24 - 3.08) | (3.35 - 4.32) | (3.18 - 4.08) | (5.21 - 5.63) | (4.37 - 6.42) |
|  |  |  |  |  |  |  |  |
| Observations |  | 139 | 139 | 139 | 139 | 139 | 139 |
|  |  | **Sensitivity analysis 5: Period (Jun/08 – Dec/19); exposure (Feb/2013)** | | | | | |
| Time |  | 0.05*** | 0.05*** | 0.06*** | 0.05*** | 0.04*** | 0.08*** |
|  |  | (0.04 - 0.07) | (0.04 - 0.06) | (0.05 - 0.08) | (0.04 - 0.07) | (0.03 - 0.05) | (0.04 - 0.11) |
| Level |  | -0.20 | 0.17 | 0.09 | -0.20 | -0.14 | -1.83*** |
|  |  | (-0.58 - 0.18) | (-0.39 - 0.73) | (-0.50 - 0.68) | (-0.66 - 0.26) | (-0.53 - 0.25) | (-2.97 - -0.70) |
| Trend |  | -0.05*** | -0.01 | -0.06*** | -0.05*** | -0.04*** | -0.05** |
|  |  | (-0.06 - -0.03) | (-0.03 - 0.00) | (-0.08 - -0.04) | (-0.07 - -0.04) | (-0.05 - -0.03) | (-0.08 - -0.01) |
| Constant |  | 4.50*** | 2.67*** | 3.88*** | 3.66*** | 5.44*** | 5.47*** |
|  |  | (4.15 - 4.86) | (2.25 - 3.09) | (3.37 - 4.38) | (3.19 - 4.12) | (5.23 - 5.65) | (4.41 - 6.53) |
|  |  |  |  |  |  |  |  |
| Observations |  | 139 | 139 | 139 | 139 | 139 | 139 |
| 95% CIs in parentheses | | |  |  |  |  |  |
| *** p<0.01, ** p<0.05, * p<0.1 | | |  |  |  |  |  |
